# Whole pancreas water T_1_ mapping at 3 Tesla

**DOI:** 10.1007/s10334-025-01224-8

**Published:** 2025-03-06

**Authors:** Elizabeth Huaroc Moquillaza, Kilian Weiss, Lisa Steinhelfer, Jonathan Stelter, Marcus R. Makowski, Rickmer Braren, Mariya Doneva, Dimitrios C. Karampinos

**Affiliations:** 1 Institute of Diagnostic and Interventional Radiology, TUM School of Medicine and Health, Technical University of Munich, Munich, Germany; 2 Philips GmbH, Market DACH, Hamburg, Germany; 3 German Cancer Consortium, a Partnership Between DKFZ and School of Medicine, Technical University of Munich, Munich, Germany; 4 Philips Innovative Technologies, Hamburg, Germany

**Keywords:** Pancreas water T_1_ mapping, Whole pancreas, Spiral trajectory, Look-Locker method, Dictionary matching

## Abstract

**Purpose:**

A fast T_1_ mapping method of the whole pancreas remains a challenge, due to the complex anatomy of the organ. In addition, a technique for pancreas water T_1_ mapping is needed, since the T_1_ is biased in the presence of fat. The purpose of this work is to accelerate the acquisition of water selective T_1_ (wT_1_) mapping for the whole pancreas at 3 T.

**Methods:**

The proposed method combines a continuous inversion-recovery Look-Locker acquisition with a single-shot gradient echo spiral readout, water-fat separation and dictionary matching for wT_1_ mapping of the whole pancreas at 3 T. The bias of T_1_ in the presence of fat was evaluated in a phantom by comparing the modified Look-Locker inversion-recovery (MOLLI) and the proposed method to MRS measurements. The present method was validated in 11 volunteers by evaluating its pancreas coverage and repeatability and by comparing it to MOLLI. Four pancreatitis patients were evaluated using the proposed method and clinical scans.

**Results:**

The phantom wT_1_ results are in better agreement to MRS (${\text{wT}}_{1} = 1.02{\text{*wT}}_{{1{\text{MRS}}}} - 47.81,{ }R^{2} = 0.99){ }$ than MOLLI (${\text{T}}_{{1{\text{MOLLI}}}} = 1.13*{\text{wT}}_{{1{\text{MRS}}}} - 74.65, R^{2} = 0.98)$. The volunteer wT_1_ results demonstrate the whole pancreas coverage capability for different fat fractions, good repeatability (${\text{wT}}_{{1,{ }2^\circ }} = 0.98*{\text{wT}}_{{1,{ }1^\circ }} + 17.40, R^{2} = 0.69)$ and lower T_1_ values than MOLLI (${\text{wT}}_{1} = 0.34{\text{*T}}_{{1{\text{MOLLI}}}} + 383.65,{\text{ R}}^{2} = 0.26)$. The wT_1_ maps in patients captured diverse pancreatitis regions with higher values $({\text{wT}}_{{1{\text{Patients}}}} = \left[ {831 - 1696} \right]\;{\text{ms}})$ than in the volunteers $\left( {{\text{wT}}_{{1{\text{Volunteers}}}} = \left[ {605 - 799} \right]\;{\text{ms}}} \right)$, thus showing their potential clinical feasibility.

**Conclusion:**

The present work proposes a wT_1_ mapping methodology of the whole pancreas at 3 T, where 24 slices ($2 \times 2 \times 5\;{\text{mm}}^{3}$) were acquired in three short breath-holds of 12 s each.

**Supplementary Information:**

The online version contains supplementary material available at 10.1007/s10334-025-01224-8.

## Introduction

Quantitative MRI provides valuable parameters for the characterization of the pancreas and the assessment of pancreatic diseases, limited if only based on qualitative MRI [1]. For instance, studies including only healthy participants have been performed to determine the normal pancreatic T_1_ value [2] and the feasibility of new quantitative techniques in the pancreas [3]. Pancreas T_1_ mapping has been mainly analyzed for organ inflammation and neoplasia. Many studies indicate that the T_1_ value of the pancreatic parenchyma increases in the presence of chronic pancreatitis (CP) [1, 4–10]. Pancreatic fibrosis is a characteristic of CP and pancreatic cancer. T_1_ would increase in CP due to a loss of acinar cells and replacement by fibrosis which originates less proteinaceous content [7]. Even at the early stages of CP, T_1_ is able to distinguish changes [4, 8, 9]; therefore, it may be a promising biomarker for this disease. Moreover, it has been found that pancreatic T_1_ correlates significantly with pancreatic exocrine function which decreases with the development of chronic pancreatic fibrosis [11]. If acquired before and after contrast, T_1_ mapping can be used to calculate a fibrosis indicator, the extracellular volume (ECV) fraction [1, 2, 6, 7, 12, 13]. A combination of quantitative parameters like ECV, proton density fat fraction (PDFF), magnetic resonance elastography (MRE) and T_1_ mapping has been also suggested to improve fibrosis diagnostic performance [7, 9]. Moreover, multiparametric mapping including T_1_ has been evaluated for CP [10] and pancreatic ductal adenocarcinoma [14].

The long duration of the T_1_ mapping methods is an impediment for their clinical use. 2D T_1_ mapping techniques like MOLLI [5, 9, 11, 12, 14], SASHA [12] and IR-SNAPSHOT [12] have been used for the evaluation of pancreatic diseases, but can estimate T_1_ in one or, as for the latter, three slices per breath-hold (10–15 s). Free-breathing MRF 2D techniques have been also designed to encode the pancreas T_1_ and T_2_ parameters of one slice in 10 s [3] to 39 s [15]. Most of the aforementioned works have used regions of interest (ROIs) in the head, tail and body of the pancreas for the T_1_ mapping analysis. The ROIs have been defined even if only three slices were available [3, 5, 9, 11, 14]. The ROIs definition might vary if the whole pancreas was covered which would also allow to assess diseases that could spread along the organ. Therefore, a whole pancreas coverage would be of advantage. Due to the diagonal orientation of the pancreas, many axial slices are needed for a detailed whole pancreas coverage which might take up to several minutes with the current 2D techniques. In contrast, 3D methods for whole pancreatic T_1_ mapping like variable flip angle (VFA) [12], dual flip angle (DFA) [4, 7, 8, 10, 16] and turbo field echo [13] sequences have been developed to acquire multiple images in a breath-hold at one second per slice as maximum. However, VFA and DFA methods require a breath-hold of long duration (~ 18 s) clearly overstraining certain patients in this population, which would compromise the quality of the T_1_ maps. Moreover, VFA and DFA methods require the acquisition of a ${\text{B}}_{1}^{ + }$ map to perform ${\text{B}}_{1}^{ + }$ correction which increments the scan time.

T_1_ is known to be biased in the presence of fat which is frequently seen in lipomatous and atrophic pancreas [17]. From the aforementioned works, only [16] evaluates the T_1_ bias by comparing pancreatic T_1_ values using 3D DFA with and without fat suppression. However, the 3D DFA T_1_ mapping technique requires a long breath-hold of 19 s and ${\text{B}}_{1}^{ + }$ correction.

Based on the previous evidence, there is an unmet need for a pancreas T_1_ mapping method with short acquisition time, with coverage of the whole pancreas, specific to the water signal and robust to ${\text{B}}_{1}^{ + }$ inhomogeneities.

This work proposes a ${\text{B}}_{1}^{ + }$-robust, rapid water T_1_ (wT_1_) mapping method at 3 T to scan the whole pancreas within 24 slices distributed in three breath-holds of 12 s each. For this purpose, the proposed method combines a continuous inversion-recovery Look-Locker (CIR-LL) acquisition, a Dixon approach and dictionary matching.

## Materials and methods

### Pulse sequence design—single slice

**Fig. 1:**
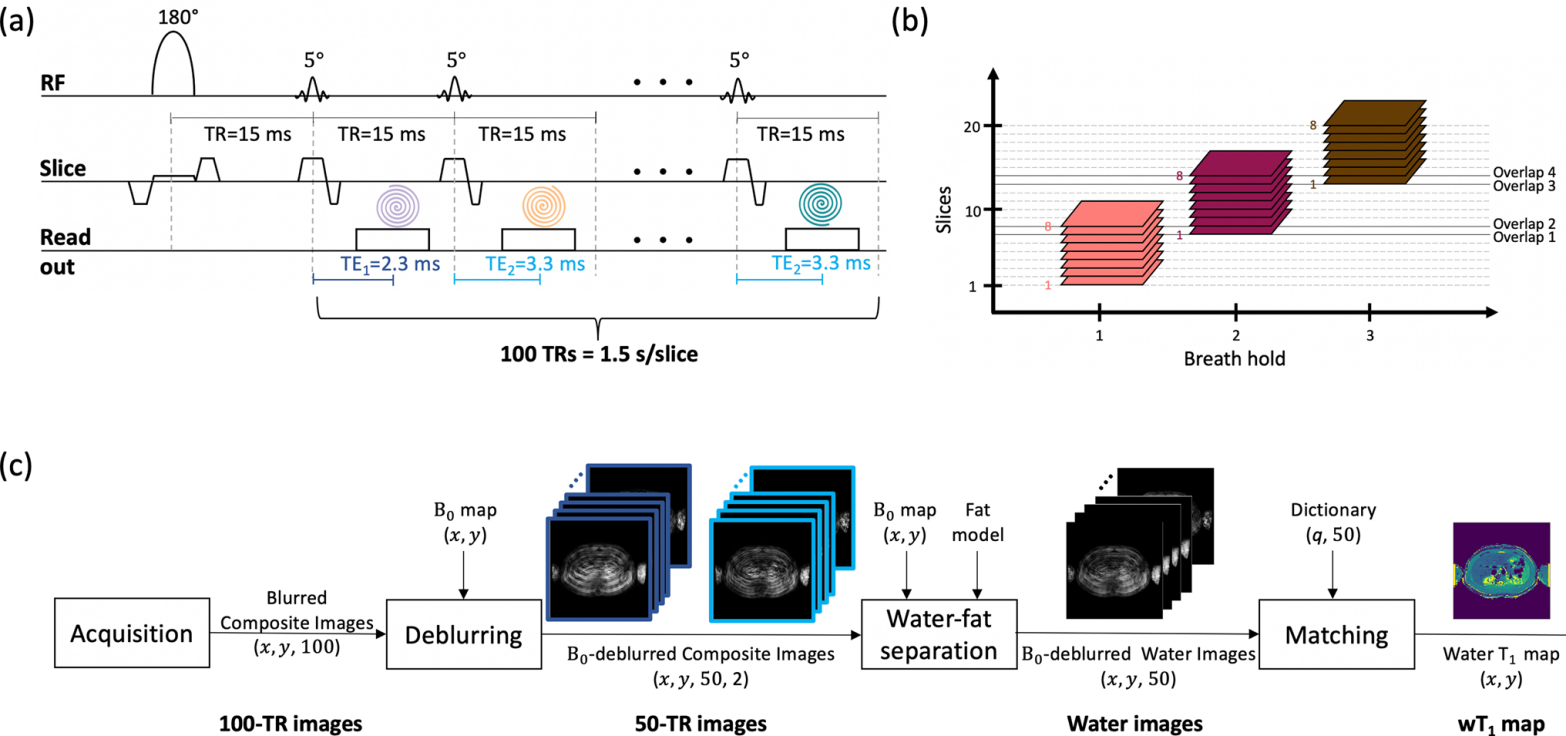
Proposed wT_1_ method. **a** Proposed single-shot continuous inversion recovery spiral sequence. **b** With the proposed sequence, eight slices (one stack) can be acquired in a breath-hold of 12 s. For the whole pancreas coverage, three separate breath-holds and an overlap of 11 mm (two slices of 5 mm thickness + a slice gap of 1 mm) between consecutive stacks is defined. **c** Block diagram to obtain the wT_1_ map of one slice. ($x, y$) represent the spatial location and 100 is the number of excitations (50 repetitions × 2 TEs). Despite the high under-sampling, the wT_1_ map presents good quality as displayed by the miniature images

A CIR-LL method for a single slice has been designed based on the ${\text{B}}_{1}^{ + }$-robust single-shot spoiled gradient echo (GRE) sequence introduced in [18] (Fig. 1a). An adiabatic slice-selective inversion pulse is followed by 5° RF pulses repeated every TR = 15 ms for 100 times. For high sampling efficiency, uniform sampling density spiral readouts are employed. A spiral readout of 9.2 ms is acquired at interleaved TE_1_/TE_2_ = 2.3/3.3 ms and is rotated (360/N_s_)° every TR for varying spatial encoding. N_s_ represents the total spiral arms needed for a fully sampled scan. Thus, the sequence allows to acquire a slice of resolution [2 × 2 × 5] mm^3^ in 1.5 s.

### Whole pancreas coverage—multiple slices

For an in vivo multi-slice acquisition, the CIR-LL sequence is repeated per slice in an interleaved manner. The slices are acquired within a breath-hold with a gap of 1 mm between two neighboring slices, which has been shown to minimize cross-talk effects [18]. Considering the difficulties of patients to hold their breath for a long time, the considered breath-hold was set to 12 s which allows the acquisition of eight slices with the proposed sequence. To cover the whole pancreas, the method was designed to acquire three stacks of eight slices. Given the possibility of organ displacement due to the separate breath-holds, an overlap of 11 mm (two slices of 5 mm thickness + a slice gap of 1 mm) was also considered for adjacent stacks. In this way, the proposed CIR-LL acquisition covers the whole pancreas in three short breath-holds of 12 s each (Fig. 1b).

### wT_1_ quantification methodology

#### Image reconstruction and main magnetic field (B_0_) deblurring

Figure 1c shows the proposed image reconstruction and wT_1_ quantification methodology. After the acquisition, the image reconstruction occurs in the scanner using gridding and coil combination techniques and producing 100 blurred composite images (100-TR images), one image per TR. Then, the images dimensions are (*x*, *y*, 100) where *x*, *y* represents the spatial location. On one hand, the images are composite since water and fat signals are superimposed. On the other hand, the images are blurred because the use of spiral trajectories in the presence of off-resonance leads to significant image blurring [19, 20]. Off-resonance originates from the B_0_ inhomogeneities or from the chemical shift, as in the case of fat. A B_0_ map is acquired as a pre-scan and is used together with the vendor software for image B_0_-deblurring on the scanner. At this point, both water and fat are still part of the total signal but the B_0_-blurring effects have been removed. The B_0_-deblurred composite images are grouped according to their TE resulting in two groups of 50 images each (50-TR images). Thus, the images dimensions are (*x*, *y*, 50, 2).

#### Water-fat separation

As a next step, the 50-TR images go through the water-fat separation process which uses a chemical species separation algorithm [21]. For this process, it is considered that every image pixel (*x*, *y*) contains two chemical species: water and fat, represented by a multi-peak fat model to account for the spectral complexity of fat [22, 23]. Given the known chemical species and using the previously acquired B_0_ map, the water-fat separation problem can be solved using signals from two different TEs as TE_1_ and TE_2_. ${\text{T}}_{2}^{*}$ effects were not considered as the proposed TEs are close to each other.

The water-fat separation process is performed for every pixel (*x*, *y*) of the 50-TR images using their two TEs. Due to the previous B_0_-deblurring, the water-fat separation step outputs 50 B_0_-deblurred water images and 50 B_0_-deblurred fat images which are still blurred due to the chemical shift. For the purpose of the present work, only the 50 water images of dimensions (*x*, *y*, 50) are used for the next step of the processing.

#### Dictionary calculation and matching

The final step to estimate the wT_1_ map of one slice is the dictionary matching. The matching process takes the water images as input to perform a complex matching using a pre-computed dictionary.

A dictionary is a set of vectors that describe the expected signal evolution for different parameters when the proposed sequence is applied. The Extended Phase Graph method [24–26] was used to compute the dictionary. The parameters for the simulation included T_1_ = [100, 3000] ms in steps of 5 ms, T_2_ = 50 ms and ${\text{B}}_{{1,{\text{ fraction}}}}^{ + }$ = 1. According to [18], a CIR-LL method with a small flip angle does not encode T_2_, is robust to ${\text{B}}_{1}^{ + }$ inhomogeneities and can be affected by slice profile effects. Therefore, T_2_ and ${\text{B}}_{1}^{ + }$ were set to specific values and the slice profile effects were modeled for the dictionary calculation [18]. To this extend, the dictionary contains vectors of 100 points because the proposed sequence considers two TEs. Therefore, every second point of the dictionary vectors was removed which is equivalent to averaging TEs. Thus, the dictionary contains complex vectors of length 50.

The matching is performed by computing the inner-product between the 50-length signal of a water pixel (*x*, *y*) and all dictionary vectors, both normalized. The T_1_ of the dictionary vector that obtains the maximum inner-product is assigned as the wT_1_ value of that specific pixel (*x*, *y*). The matching is repeated for every pixel (*x*, *y*) of the water images obtaining the wT_1_ map.

### MR measurements and analysis

Phantom and in vivo measurements were performed on a 3 T Ingenia Elition X scanner (Philips Healthcare, Best, the Netherlands) with the sequence parameters summarized in Table 1. Manual segmentations were drawn in the different images for evaluation using ITK-SNAP [27].

**Table 1:**
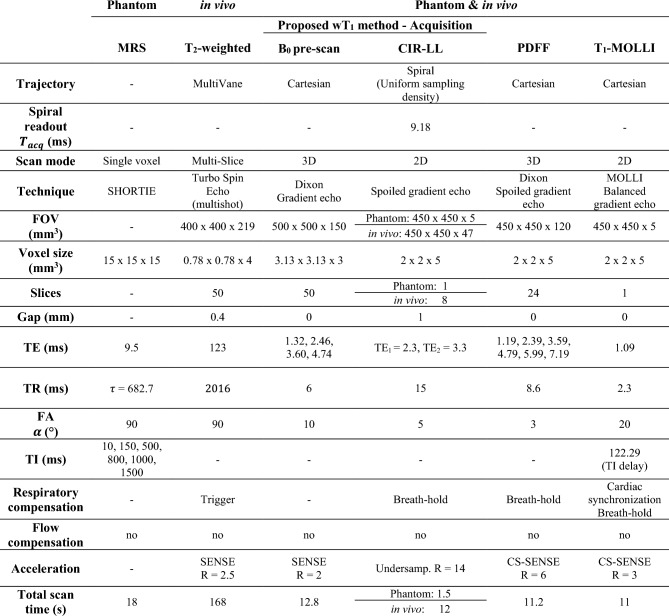
Sequence parameters used in the phantom and in vivo experiments. The sequences included the MRS method as reference, T_2_-weighted imaging for anatomical guidance, the acquisitions needed for the proposed wT_1_ method, containing the B_0_ pre-scan and the proposed CIR-LL method, and the PDFF and T_1_-MOLLI maps for comparison

#### Phantom measurements

A phantom with vials of variable PDFF and wT_1_ values (Calimetrix, Madison, WI, USA) was used to validate the proposed method. The phantom contains emulsions of peanut oil and doped water in agar form in 12 vials distributed as shown in Fig. S1.

The phantom scan started with the PDFF maps acquisition to evaluate the fat fraction in the vials. Afterwards, the T_1_-MOLLI map was acquired to estimate the T_1_ value of the emulsions of water and fat in each vial. Then, the B_0_ maps were acquired and were adapted to the FOV and voxel size of the wT_1_ map. Finally, the proposed CIR-LL method was executed obtaining the 100-TR images. The 100-TR images were processed with the proposed workflow (Fig. 1c) to estimate the one-slice wT_1_ map of the phantom using the corresponding B_0_ map, a 10-peak fat model specific to peanut oil [22] and the previously defined dictionary.

In order to obtain wT_1_ reference values, single-voxel short-TR multi-TI multi-TE STEAM MRS measurements [28] were performed at the center of every phantom vial. MRS data were processed with a time-domain-based fitting approach using the ALFONSO [29] framework. The MRS processing framework includes coil combination, signal averaging, frequency offset correction, phase correction and signal model fitting to estimate T_1_. The signal model fitting was performed in the time domain and jointly for the TI series of the water peak of the spectrum using the nonlinear least-squares solver NL2SOL [28, 30]. ROIs were also defined in the T_1_-MOLLI and wT_1_ maps for all vials. To evaluate the proposed method and MOLLI with respect to MRS, the average of the ROIs defined on both maps were compared to the wT_1_-MRS values.

#### In vivo measurements

11 volunteers and four pancreatitis patients participated in the in vivo measurements. The experiments were performed using the scanner’s built-in 12-channel posterior coil and a 16-channel anterior coil. The patient cohort included four individuals diagnosed with necrotizing pancreatitis per documentation in the local hospital information system. In three of the cases, acute pancreatitis was attributed to alcohol consumption, while one case developed in the context of chronic autoimmune pancreatitis. Ethical approval from the local ethics commission was obtained prior to conducting the study and all participants provided informed consent.

For all volunteers, T_2_-weighted images were acquired first and were used to plan the next pancreas scans. The B_0_ maps were acquired in free-breathing and were adapted to the FOV and voxel size of the wT_1_ maps. Then, the proposed CIR-LL acquisition was performed during a 12 s breath-hold covering eight pancreas slices. The acquisition occurred in an interleaved way (slice 1, 4, 7, 2, 5, 8, 3, 6) and without delay between slices. For each slice, the acquired 100-TR images were processed with the proposed pipeline (Fig. 1c) to estimate the wT_1_ maps using the B_0_ maps, a 7-peak fat model [23] and the specified dictionary. For the coverage of the whole pancreas, the B_0_ pre-scan and the proposed CIR-LL acquisition were performed for three consecutive stacks. Each stack comprises eight slices and an overlap of two slices between the consecutive stacks was considered. The three-stack B_0_ pre-scan and CIR-LL acquisition were performed twice to analyze the repeatability of the present work. Moreover, to evaluate the variation of fat content in the pancreas, the PDFF maps were acquired in a breath-hold. Finally, the T_1_-MOLLI acquisition of one slice was performed, up to three times, using cardiac synchronization in a breath-hold.

The T_2_-weighted images were used also as anatomical guidance for the manual segmentation of the pancreas in the volunteers’ wT_1_, T_1_-MOLLI and PDFF maps. The kidneys and liver were the main anatomical landmarks to determine the correspondence between the slices of the different maps. In this way, the pancreas was segmented in 2D for all slices of the three-stack wT_1_ maps, obtaining a 3D whole organ segmentation. For the one-slice T_1_-MOLLI maps, the pancreas was manually segmented in 2D in the slice. Four cases that depict the PDFF variation were selected. For these cases, the pancreas segmentation was performed on the PDFF maps corresponding to the T_1_-MOLLI maps, adjusting the contrast when necessary. To evaluate the importance of the water-fat separation step and the dependency of T_1_ to TE [31], the 50-TR images of the volunteer with the highest PDFF value in the pancreas were analyzed. The 50-TR images corresponding to each TE were used directly for the matching processing, ignoring the water-fat separation step. Thus, a T_1_ map from the composite signal (T_1comp_), including water and fat, was estimated for TE_1_ and TE_2_. Moreover, T_1comp_ simulations using the proposed method were performed for wT_1_ in the range [600, 1700] ms and PDFF in the range [5, 40]%, observed as the fat content range including pancreatic diseases [16] and pancreatitis [10]. To evaluate the repeatability, the average wT_1_ value of the segmented 3D pancreas region in each stack was compared between the first and the second measurement. Furthermore, the wT_1_ map corresponding anatomically to the T_1_-MOLLI map was identified in order to compare the average of the 2D segmentations on both maps.

For the patients, T_2_-weighted images, PDFF, B_0_ and wT_1_ maps were performed similar as for the volunteers. However, only one measurement of the three-stack B_0_ pre-scan and the proposed CIR-LL method was acquired to estimate the wT_1_ maps. To test clinical applicability, the acquired images were compared to contrast-enhanced CT images at the arterial and portal phase acquired as part of the clinical routine imaging of each patient. To evaluate the PDFF, wT_1_ and CT values in the presence of pancreatitis, circular ROIs (diameter varying from 6.5 to 17.4 mm according to the anatomy) were defined in the pancreas head, body and tail in the PDFF maps, wT_1_ maps and in the CT images for all patients. The average wT_1_ value of the 3D stack segmentations in the volunteers and the wT_1_ values of the ROIs in the pancreatitis patients were then compared to observe their differences.

## Results

### Phantom results

**Fig. 2:**
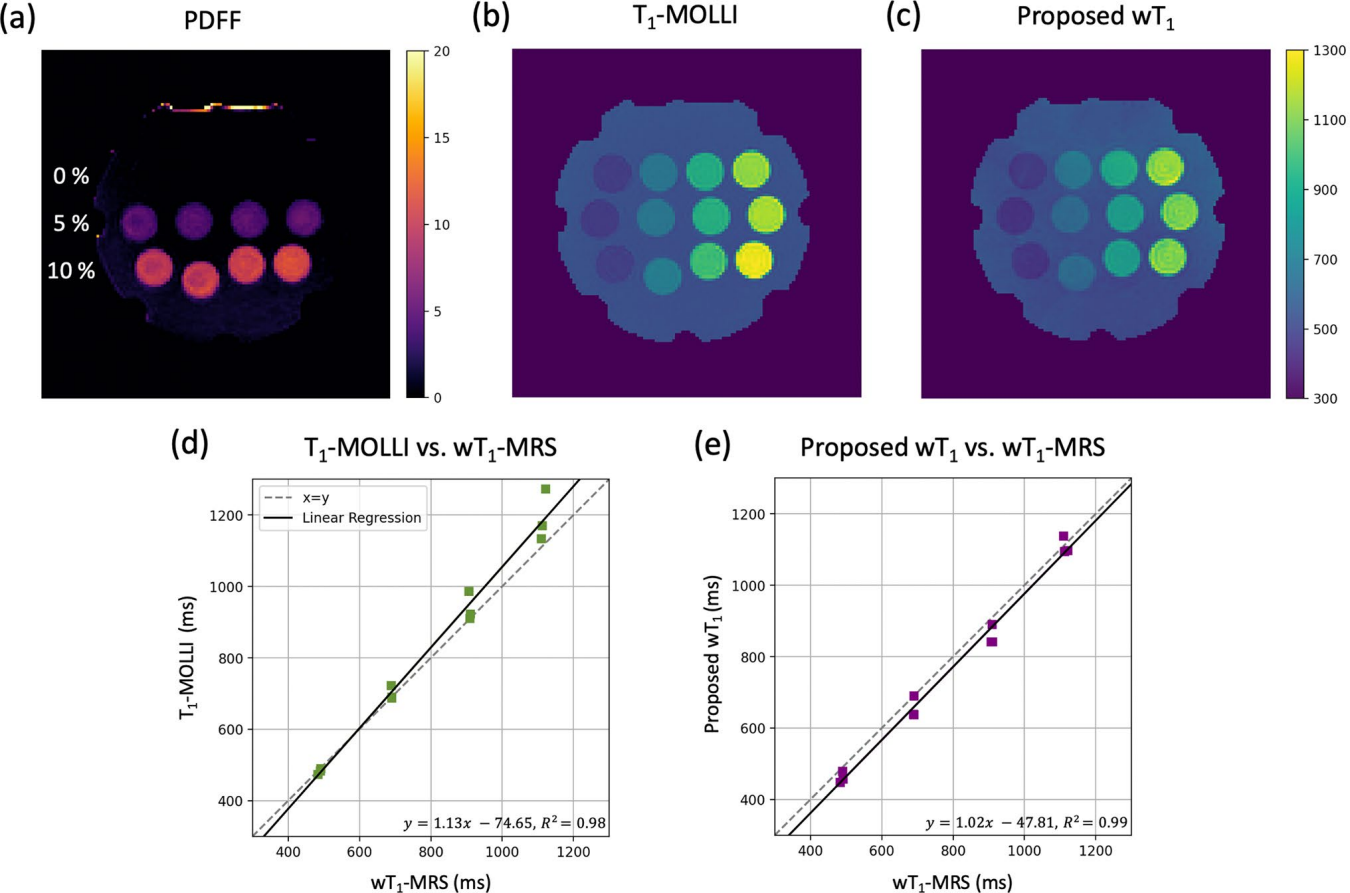
Phantom measurements. **a** PDFF map of the phantom confirms that the vials are distributed in rows of 0%, 5% and 10% PDFF. **b** T_1_-MOLLI map shows that the vials in the same column have similar T_1_ values. **c** Proposed wT_1_ map presents the same T_1_ distribution as T_1_-MOLLI, but lower values. **d** Average of the ROIs defined in T_1_-MOLLI compared to wT_1_-MRS. **e** Average of the ROIs defined in the proposed wT_1_ compared to wT_1_-MRS

The acquired PDFF map (Fig. 2a) confirmed the fat content variation in the phantom vials which are organized in rows of 0%, 5% and 10% PDFF. Moreover, the T_1_-MOLLI map (Fig. 2b) corroborated that the T_1_ values of the vials are different along the rows, but similar along the columns. However, it is known that T_1_ can be biased in the presence of fat. Therefore, it was expected that the proposed wT_1_ map (Fig. 2c) presented different values relative to the T_1_-MOLLI map which estimates the T_1_ values from a signal containing water and fat.

The average of the ROIs defined on the T_1_-MOLLI and wT_1_ maps were compared to the wT_1_-MRS values as shown in Fig. 2d–e. The proposed wT_1_ method is in better agreement with wT_1_-MRS (${\text{wT}}_{1} = 1.02{\text{*wT}}_{{1{\text{MRS}}}} - 47.81,{ }R^{2} = 0.99)$ than T_1_-MOLLI (${\text{T}}_{{1{\text{MOLLI}}}} = 1.13*{\text{wT}}_{{1{\text{MRS}}}} - 74.65, R^{2} = 0.98)$ showing that 100 TRs can encode the T_1_ recovery curve. In general, wT_1_ values are lower than T_1_-MOLLI values. The results of the phantom’s ROI analysis are listed in Table S1.

### In vivo results

**Fig. 3:**
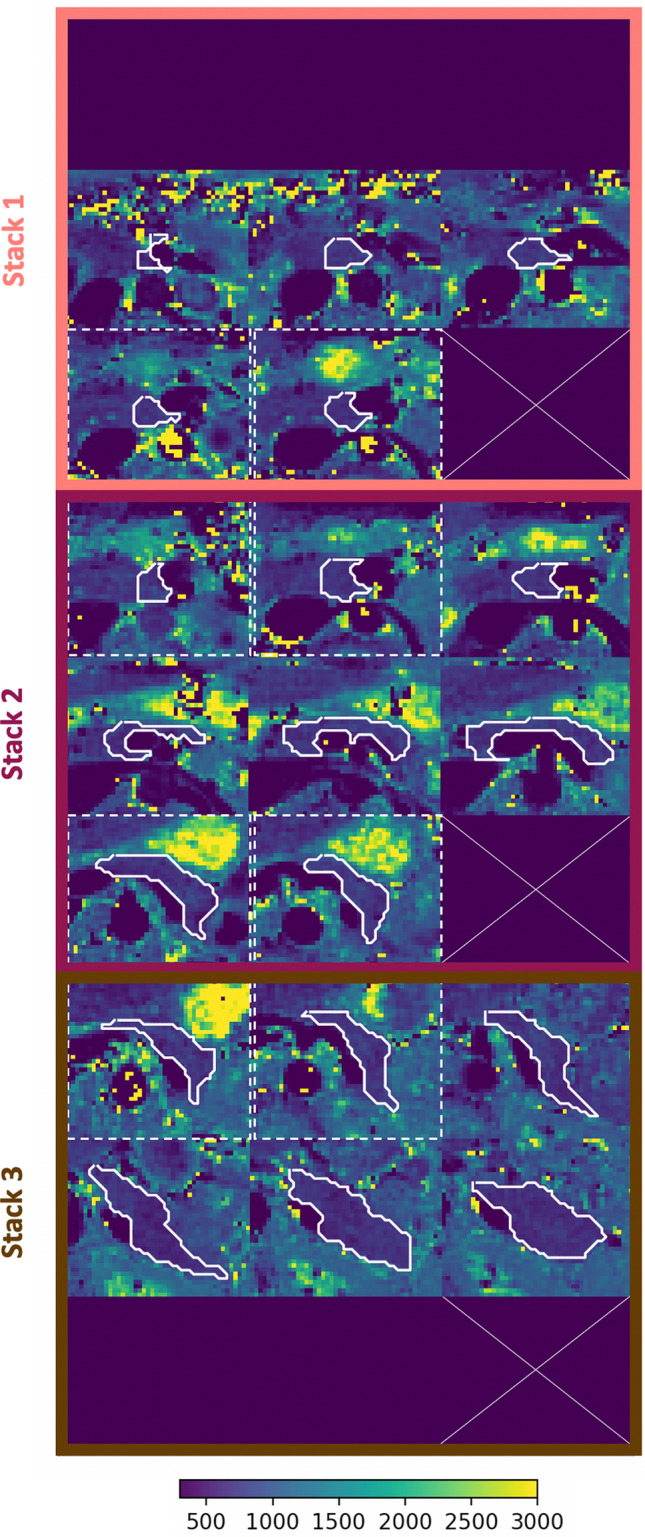
Proposed wT_1_ maps in a volunteer. Zoomed wT_1_ maps of the whole pancreas. Three stacks of eight slices were acquire for whole pancreas coverage. Each stack, acquired in a breath-hold, is presented in a color box. Slices which did not contain pancreas parenchyma are shown in dark blue. The pancreas is highlighted with a white contour on each slice. Dotted white boxes group vertically the slices in an overlap. Corresponding slices in an overlap show similar anatomical structures. Fig. S2 presents the complete wT_1_ maps

The proposed method covers the whole pancreas. Despite the different breath-holds and the risk of organ displacement, the two-slice-overlap defined between stacks results in minimal variations of the pancreas anatomy. For one volunteer, Fig. 3 presents a zoomed version of the proposed wT_1_ maps focused on the pancreas while Fig. S2 presents the complete wT_1_ maps. For all volunteers, Fig. S3 shows the proposed wT_1_ map of the middle slice and the corresponding segmentation highlighting the good performance of the method despite the heterogeneous pancreatic anatomy.

**Fig. 4:**
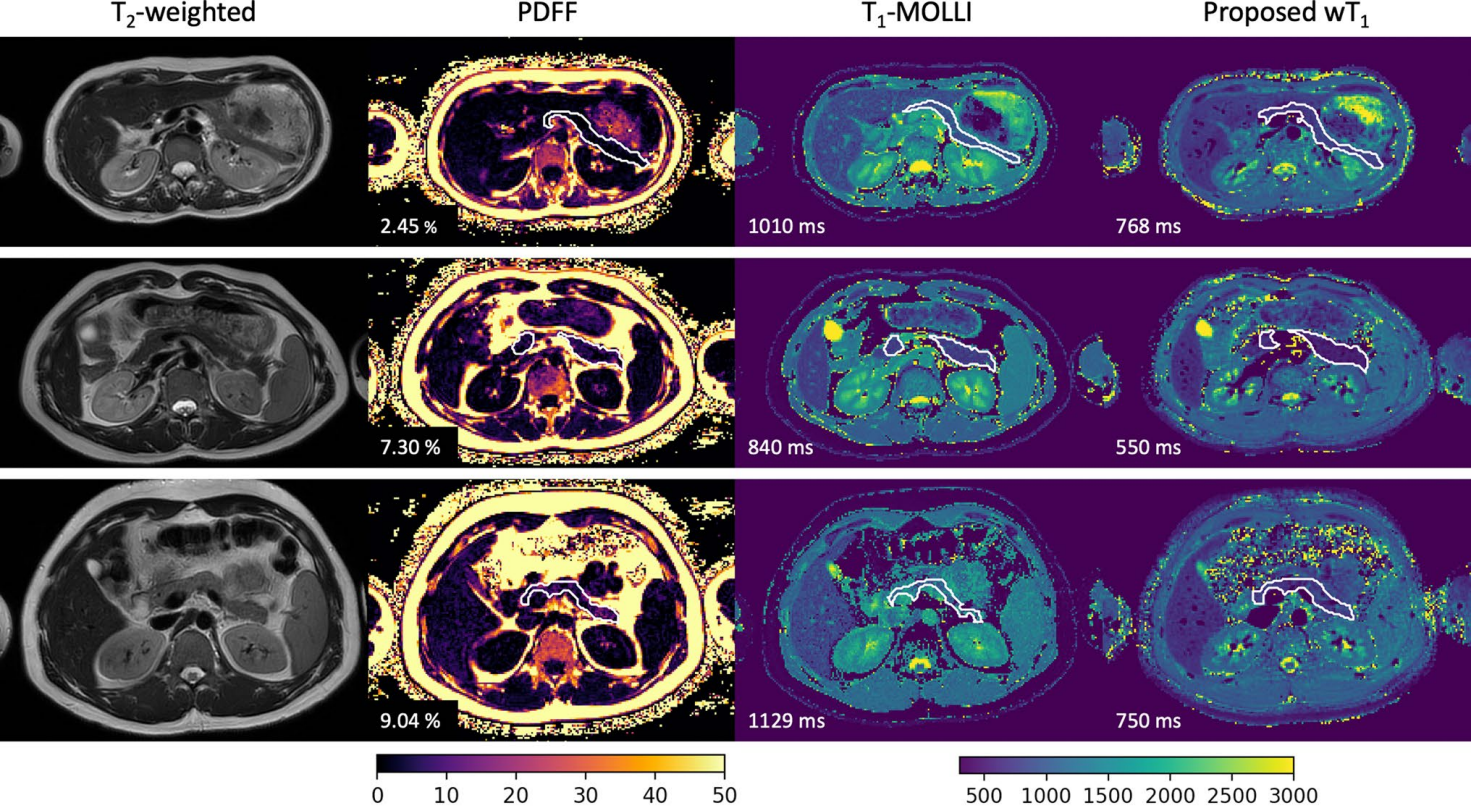
In vivo measurements in volunteers. T_2_-weighted images, PDFF, T_1_-MOLLI and the proposed wT_1_ maps are organized in columns. Every row corresponds to one volunteer. The manual segmentation of the pancreas is displayed in each map in white. The average of the segmented region is shown in the bottom left corner of the images. The PDFF maps present different values for all the cases which shows the applicability of the proposed method in the presence of different fat concentrations. In comparison to the proposed wT_1_ maps, the T_1_-MOLLI maps present higher values in the pancreas and in the vessels, clearly visible in the liver

The pancreas parenchyma is a complex structure whose PDFF, T_1_-MOLLI and wT_1_ values vary within the organ. The proposed wT_1_ method depicts the pancreas comparable to anatomical T_2_-weighted images and is applicable to different pancreas PDFF values. Moreover, the wT_1_ maps present lower values in comparison to the T_1_-MOLLI maps. In addition, both methods present differences within the vessels. Figure 4 presents a comparison of the aforementioned images.

**Fig. 5:**
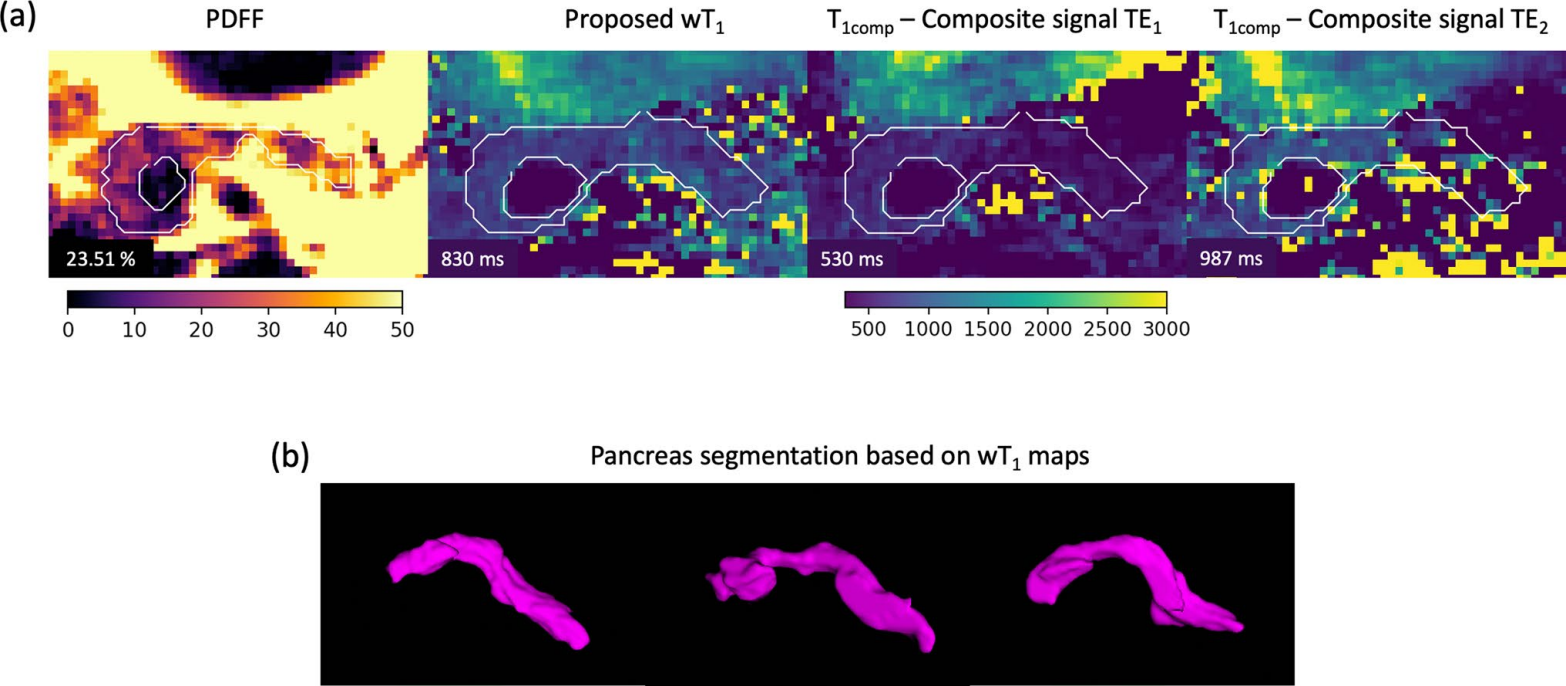
Highest PDFF case and pancreas segmentation. **a** PDFF, proposed wT_1_ and T_1comp_ map for TE_1_ and TE_2_ in the volunteer with highest PDFF in the pancreas. The 50-TR images of each TE were matched directly to the dictionary to obtain the T_1comp_ maps. The pancreas was segmented manually in all maps as displayed in white. The segmentation’s mean value is shown in the bottom left corner of every image. Despite the high PDFF value, the volunteer does not present any pathology. Due to the presence of fat and the selection of TE, the T_1comp_ maps present different results between them and also differ from the wT_1_ map highlighting the need of a water-fat separation approach. **b** The 3D visualization of the pancreas is possible due to the whole pancreas coverage of the proposed method

Figure 5a shows the evaluation of the importance of the water-fat separation step in the pancreas region and the dependency of T_1_ on the TE in the presence of fat [31]. In a pancreas region with high PDFF, if no water-fat separation is considered, the T_1comp_ at TE_1_ and TE_2_ presents lower and higher values than the proposed wT_1_, respectively. The latter is in agreement with the T_1comp_ simulations displayed in Fig. S4. The simulations show that for a PDFF in the range [5, 40]% the wT_1_ variation is on the range [− 41.47, 80.71]% which confirms the importance of the water-fat separation step.

Given the whole pancreas coverage offered by the proposed method, the segmentation of the pancreas parenchyma in all wT_1_ maps allows a smooth 3D visualization [27] of the whole organ (Fig. 5b).

**Fig. 6:**
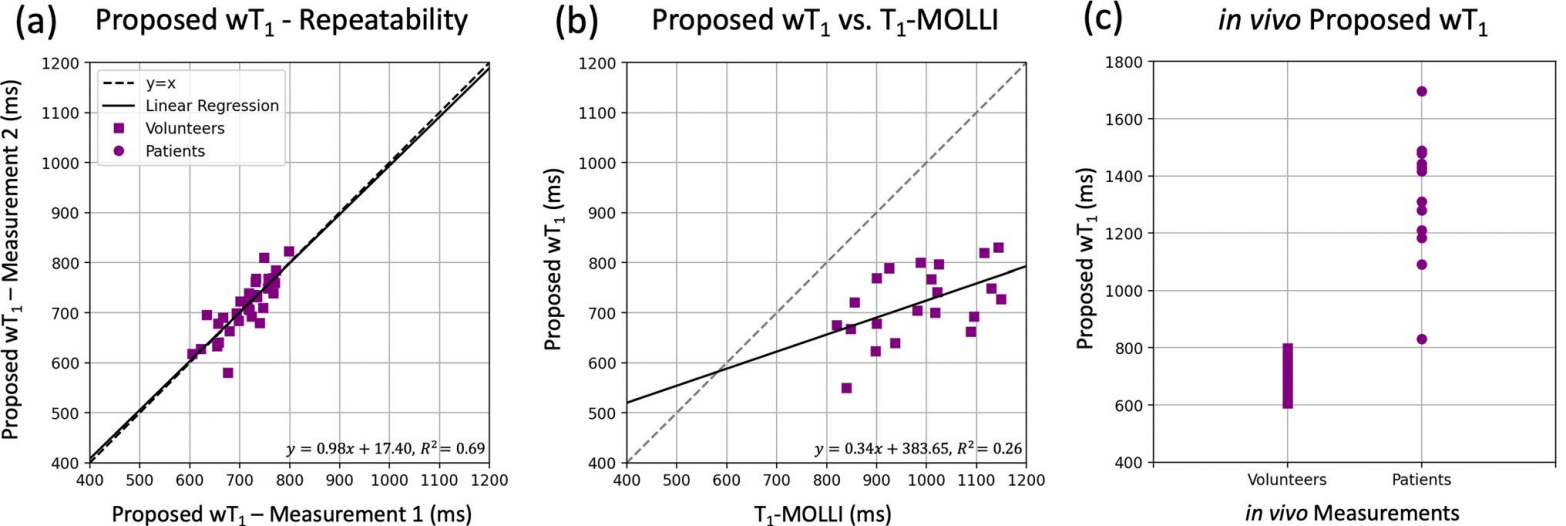
In vivo quantitative analysis. **a** The mean values of the 3D pancreas wT_1_ segmentations in all stacks were compared between the two consecutive measurements of the proposed method in the volunteers, showing repeatability. **b** The mean values of the 2D segmentations on the T_1_-MOLLI maps and their corresponding wT_1_ maps were compared. The proposed method presents lower values than MOLLI. **c** The wT_1_ mean values of the 3D segmentation in each stack in the volunteers (Measurement 1, $\mathcal{N}\left( {713.8,{ }46.7} \right)$) were compared to the wT_1_ mean values obtained from the ROIs drawn in the head, body and tail of the pancreas for the pancreatitis cases ($\mathcal{N}\left( {1321.1,{ }215.2} \right)$). All wT_1_ mean values corresponding to the patients are higher than for the volunteers

A quantitative evaluation was performed in volunteers and patients. The analysis of the volunteers’ pancreas segmentation from the two three-stack measurements demonstrates the repeatability (${\text{wT}}_{{1,{ }2^\circ }} = 0.98*{\text{wT}}_{{1,{ }1^\circ }} + 17.40, R^{2} = 0.69)$ of the proposed method (Fig. 6a). The ROI evaluation also confirms that the wT_1_ values are lower than their corresponding T_1_-MOLLI values (${\text{wT}}_{1} = 0.34{\text{*T}}_{{1{\text{MOLLI}}}} + 383.65,{\text{ R}}^{2} = 0.26)$ for all the cases (Fig. 6b), as shown in Fig. 4 and previously in the phantom results. Furthermore, the ROI analysis in the patients with pancreatitis showed that the wT_1_ values are higher in the presence of acute inflammation than in all volunteers (Fig. 6c), which is in accordance with previous studies [5]. The ROI values used for the repeatability analysis and the comparison between the proposed wT_1_ and T_1_-MOLLI for the volunteers are listed in Table S2 and Table S3, respectively. The ROIs values from the PDFF maps, wT_1_ maps and CT images of the four pancreatitis cases are summarized in Table S4.

**Fig. 7:**
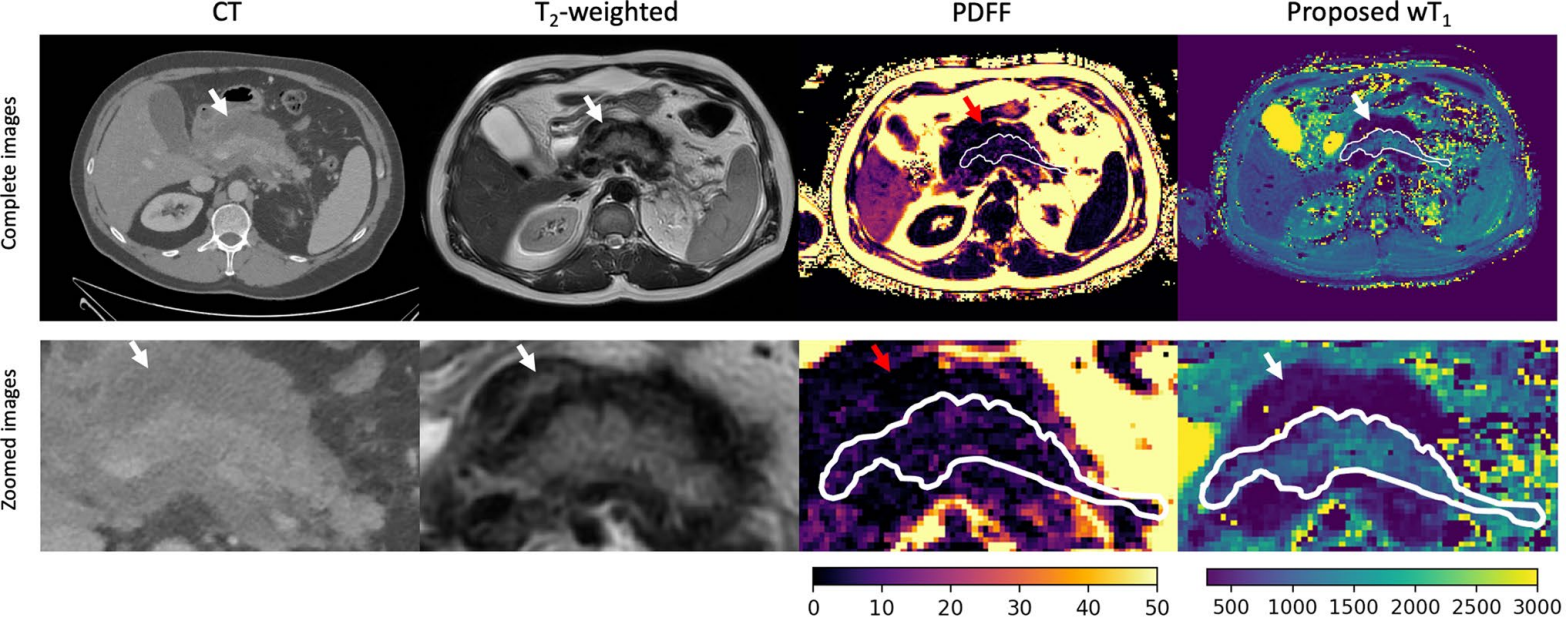
Comparison between clinical images and wT_1_ maps for a necrotizing pancreatitis case. A 46-year-old woman presented alcohol-induced necrotizing pancreatitis. As part of the clinical routine imaging of the patient, an axial contrast-enhanced CT scan during the portal phase was acquired. T_2_-weighted images, PDFF and wT_1_ maps were acquired on the same day as the CT scan. The displayed CT image, T_2_-weighted image and PDFF map demonstrate heterogeneous enhancement of the pancreatic parenchyma with poorly defined contours, surrounded by a heterogeneous fluid collection suggestive of walled-off necrosis (arrow). Moreover, the pancreas parenchyma (white contour) exhibits fat content as displayed by the PDFF map. This underscores the importance of the water-fat separation in accurately estimating T_1_ values in the pancreas. Remarkably, the wT_1_ map provides a detailed depiction of the pancreatic anatomy, differentiating necrotic tissue from healthy parenchyma with greater contrast than both the CT image and the PDFF map

**Fig. 8:**
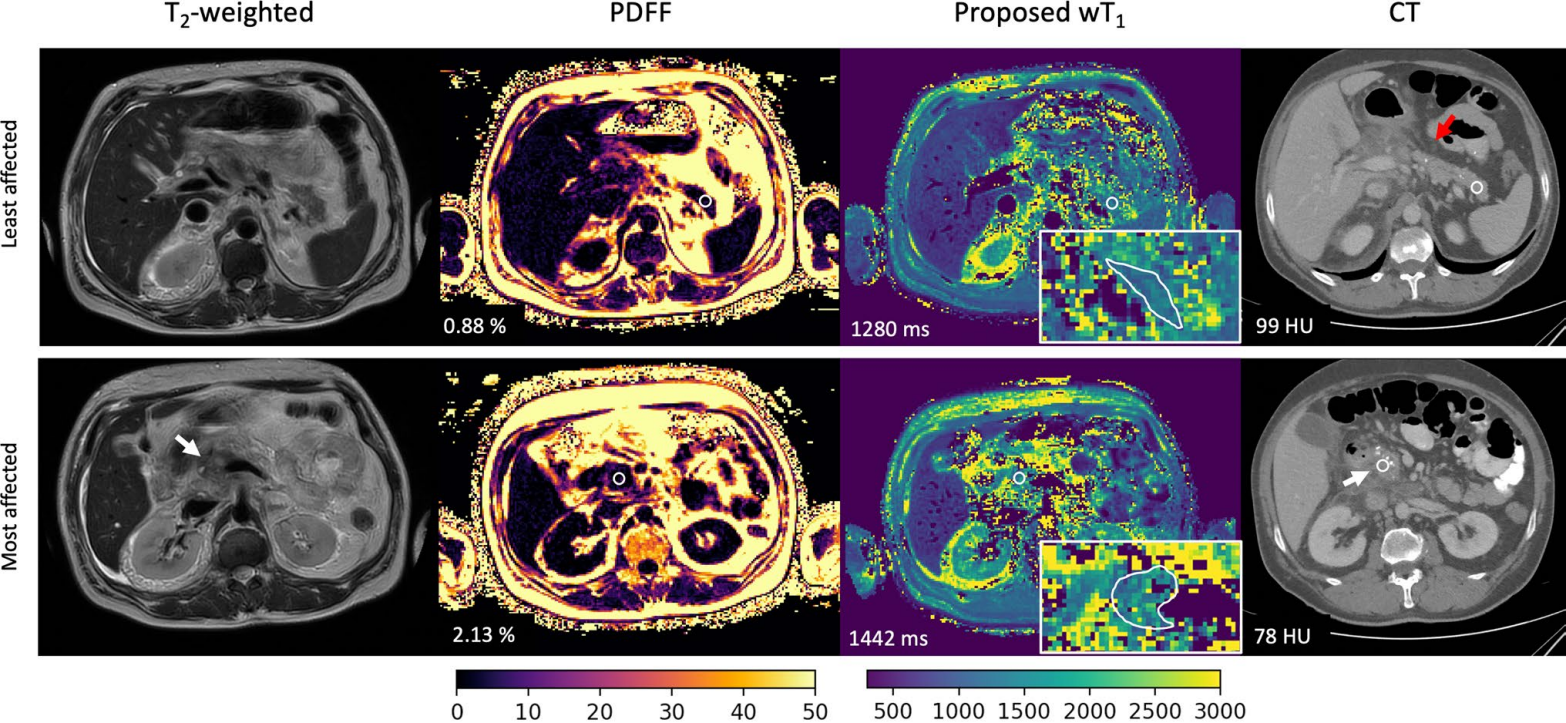
Differentiation of regions affected by pancreatitis in clinical images and wT_1_ maps. A 52-year-old man with a history of alcohol abuse was hospitalized due to necrotizing pancreatitis. T_2_-weighted images, PDFF maps, and CT images were compared to the proposed wT_1_ maps. The CT images, acquired one week earlier during the arterial and venous phases, reveal that the tail of the pancreas (first row) is the region least affected by the pancreatitis, while the head of the pancreas (second row) is the most affected. The images show pancreatic swelling, predominantly in the head (white arrows), characterized by heterogeneous and reduced enhancement, along with fluid collection in the peripancreatic area. Mild peripancreatic fat stranding is also evident (red arrow), particularly near the body and tail of the gland. ROIs (white circles) indicate a higher PDFF value in the most affected areas, confirming the presence of fat within the pancreatic parenchyma. Additionally, the ROIs show that the wT_1_ maps provide differentiation between the least and most affected regions as depicted by the CT Hounsfield unit (HU) values

The proposed method clearly depicts the changes in the pancreas parenchyma due to pancreatitis. In the wT_1_ maps, diseased areas, including areas with necrosis, are well delineated, on par or even more clearly compared to respective PDFF maps and CT images (Fig. 7). Moreover, the PDFF map proves the presence of fat in the pancreas parenchyma which would affect the estimation of T_1_ in the absence of water-fat separation. Figure 8 shows the PDFF, wT_1_ and CT images of two pancreas regions differently affected by pancreatitis. As the CT ROIs values, the wT_1_ ROIs values also allow to differentiate the least and the most affected pancreas region.

## Discussion

The present work proposes a wT_1_ mapping methodology covering the whole pancreas at 3 T. 24 slices (2 × 2 × 5 mm^3^) are acquired in three short breath-holds of 12 s each, allowing the whole coverage of this heterogeneous organ. The in vivo measurements showed that the pancreas can present different fat concentrations in its structures highlighting the need of water-fat separation for pancreatic T_1_ estimation. In this regard, the proposed method offers a solution to estimate the pancreatic T_1_ values without fat infiltration.

The water-fat separation step of the proposed pipeline is fundamental for the estimation of T_1_ in the pancreas. The in vivo PDFF maps have shown the variation of the fat concentration within the pancreas while Fig. S4 and Fig. 5a have shown how the T_1_ estimation can vary due to the content of fat and the selection of TE. The proposed method offers a solution to remove the T_1_ bias in the presence of fat by performing the water-fat separation step and focusing on wT_1_. As shown in Fig. 1c, it is possible to isolate the B_0_-deblurred water signal because the proposed pipeline includes a deblurring step which takes care of the off-resonance due to B_0_ inhomogeneities that affects the water signals.

The proposed method presents major improvements compared to current techniques for pancreas T_1_ mapping. The water-fat separation step distinguishes the proposed method from the majority of current techniques as a fat-suppressed pancreas T_1_ mapping method has been addressed only by [16]. In particular, in comparison to the free-breathing MRF methods [3, 15], the present work allows for a multi-slice acquisition and ensures the robustness to ${\text{B}}_{1}^{ + }$ inhomogeneities. Furthermore, compared to other methods using breath-holds up to 19 s [4, 8, 16], the proposed work considers a shorter breath-hold for a better patient compliance.

For the phantom and in vivo measurements, T_1_-MOLLI presented higher values in comparison to the proposed method. The presence of fat might explain these differences because MOLLI uses the composite signals to estimate T_1_, while the proposed method uses the water signals. Moreover, the MOLLI sequence [32] starts with a non-slice-selective inversion pulse exciting the whole abdominal volume. Therefore, the blood from other parts of the volume is present in the acquired slice as shown in the vessels in the T_1_-MOLLI maps. The blood has its own T_1_ [33] which together with the T_1_ of fat can alter the estimation of the T_1_ of the pancreatic tissue. Furthermore, MOLLI relies on a whole breath-hold for the acquisition of one slice. If the breath-hold duration is long, the acquisition is prone to movement or respiratory artifacts that can alter the T_1_ estimation.

The wT_1_ values in the patients, higher than in the volunteers, and the repeatability of the method confirmed its clinical potential for the assessment of pancreatitis in the presence of fat. Especially because the fat content can vary in the different pancreas regions as shown in Table S4. Acutely diseased pancreatic regions were well delineated and presented higher wT_1_ values compared to normal parenchyma, as expected from inflamed, edematous tissue. Thus, a potential application of our sequence would include the detection and treatment monitoring of patients suffering from type 2 autoimmune pancreatitis (AIP) with multifocality and young patient age strongly arguing for MR-based monitoring.

For three pancreatitis cases, the analyzed CT images were acquired two, six and seven days before the wT_1_ maps which is not ideal for an accurate comparison of the Hounsfield unit (HU) and wT_1_ values. Therefore, the CT images were used to identify affected organ regions without aiming to find a direct relation between the wT_1_ and HU values. In this way, it has been shown that, as the CT values, the wT_1_ values can distinguish regions differently affected by pancreatitis. This is promising towards a non-invasive ionizing-radiation-free assessment of pancreatitis-induced spatial heterogeneity changes of the pancreas using the proposed method. However, a larger scale study, including simultaneous CT images and a whole routine MRI protocol with diffusion weighted MRI and dynamic contrast-enhanced T_1_-weighted images, is needed to further evaluate the proposed method for the characterization of pancreatitis-induced pancreas changes.

This work presents some limitations. First, the proposed resolution of [2 × 2 × 5] mm^3^ might be still thick for some pancreas applications as the pancreas is a small organ. The proposed resolution was found suitable as the present work evaluates wT_1_ in the pancreas regions in the context of metabolic changes originated by pancreatitis. However, in the context of pancreatic lesions, thinner slice thickness than the proposed 5 mm might be of advantage. The sequence can be adapted to a slice thickness thinner than 5 mm, but more spiral arms would be needed to compensate the SNR which would extend the scan time. Second, the B_0_ maps are acquired as a pre-scan and in free-breathing which requires extra time and might cause misregistration with respect to the images acquired by the proposed CIR-LL sequence. The present work can be improved by acquiring the B_0_ maps in a breath-hold or acquiring more TEs to estimate B_0_ from the data [19, 34]. Third, although the breath-hold duration is short and did not represent a problem for the presented in vivo measurements, it can be still difficult for patients to comply with it. If the breath is not held adequately, not only the image quality might be affected, but also the stacks could have a mismatch affecting the pancreas coverage. The short slice acquisition time of 1.5 s suggests that the proposed method can be adapted to respiratory triggered acquisitions. However, a further analysis of the need of motion correction and the use of a slice-selective inversion pulse without a breath-hold would be needed. Fourth, the processing uses image space data and a dictionary without compression. Although no undersampling artifacts were noticed in the wT_1_ maps, the use of advanced k-space reconstruction and water-fat separation algorithms [31, 35] can be of advantage. Moreover, the dictionary contains redundant information in its original form; therefore, SVD compression [36], low-rank reconstruction [37–39] or deep learning [40] methods could be used to further improve the quality of the maps or accelerate the acquisition. Fifth, ${\text{T}}_{2}^{*}$ was not included in the water-fat model used for water-fat separation. The proposed method includes only 2 TEs from which ${\text{T}}_{2}^{*}$ cannot be estimated. ${\text{T}}_{2}^{*}$ can cause blurring along the spiral read out. This particularly affects patients with pancreatic iron deposition [1]. For these cases, the spiral read out might need to be shortened increasing the undersampling factor. The extension to more TEs for ${\text{T}}_{2}^{*}$ estimation can be investigated in future work.

## Conclusion

The present work proposes a wT_1_ mapping methodology of the whole pancreas at 3 T: 24 slices (2 × 2 × 5 mm^3^) were acquired in three short breath-holds of 12 s each. The proposed method presents a water-fat separation step as a solution to the T_1_ bias in the pancreas due to the presence of fat and has shown differentiation between pancreatitis cases and volunteers.

## Supplementary Information

Below is the link to the electronic supplementary material.

Supplementary file1 (DOCX 7072 KB)

## Data Availability

The datasets generated and/or analyzed during the current study are not publicly available due privacy or ethical restrictions, but are available from the corresponding author on reasonable request.

## Abbreviations

MRI: Magnetic resonance imaging
${\text{B}}_{1}^{ + }$: Transmit radiofrequency field
FA: Flip angle
VFA: Variable flip angle
GRE: Gradient echo
LL: Look-Locker
TI: Inversion time
MOLLI: Modified Look-Locker inversion-recovery
CIR-LL: Continuous inversion-recovery Look-Locker
MRF: Magnetic resonance fingerprinting
wT_1_: Water T_1_
RF: Radiofrequency
B_0_: Main magnetic field
PDFF: Proton density fat fraction
SHORTIE: Short-TR multi-TI multi-TE
MRS: Magnetic resonance spectroscopy
FOV: Field of view
ROI: Region of interest
SD: Standard deviation
HU: Hounsfield unit
T: Tesla
