# Supplementary material for: Whole pancreas water T1 mapping at 3 Tesla
Source: MAGMA. 2025 Mar 6;38(2):271–83. doi: 10.1007/s10334-025-01224-8 (PMC11913948; doi:10.1007/s10334-025-01224-8)
Supplement: Supplementary file 1 — Supplementary file1 (DOCX 7072 KB) [file 10334_2025_1224_MOESM1_ESM.docx]

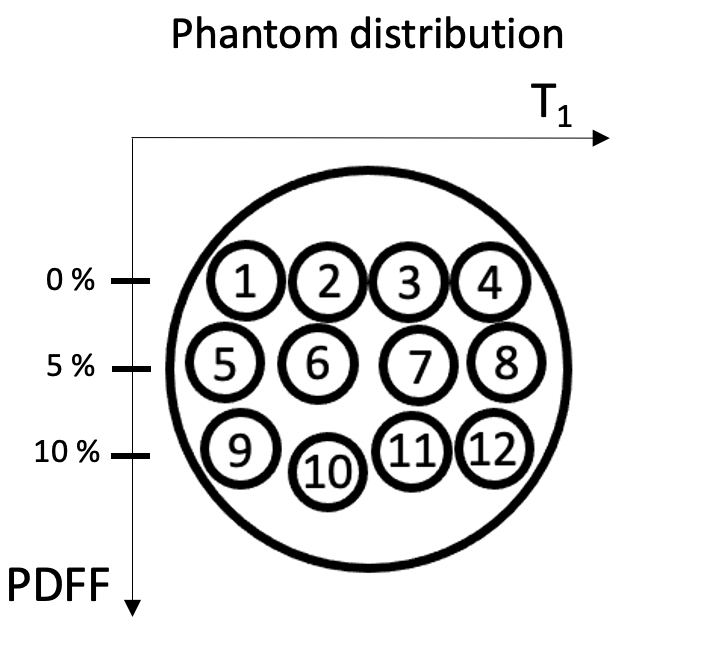


**Fig. S1** **Phantom’s vials distribution.** The Phantom (Calimetrix, Madison, WI, USA) includes 12 vials distributed in three rows and four columns. Each row groups vials with a different PDFF (0%, 5% and 10%). Vials that belong to the same column have similar T_1_ values.

**
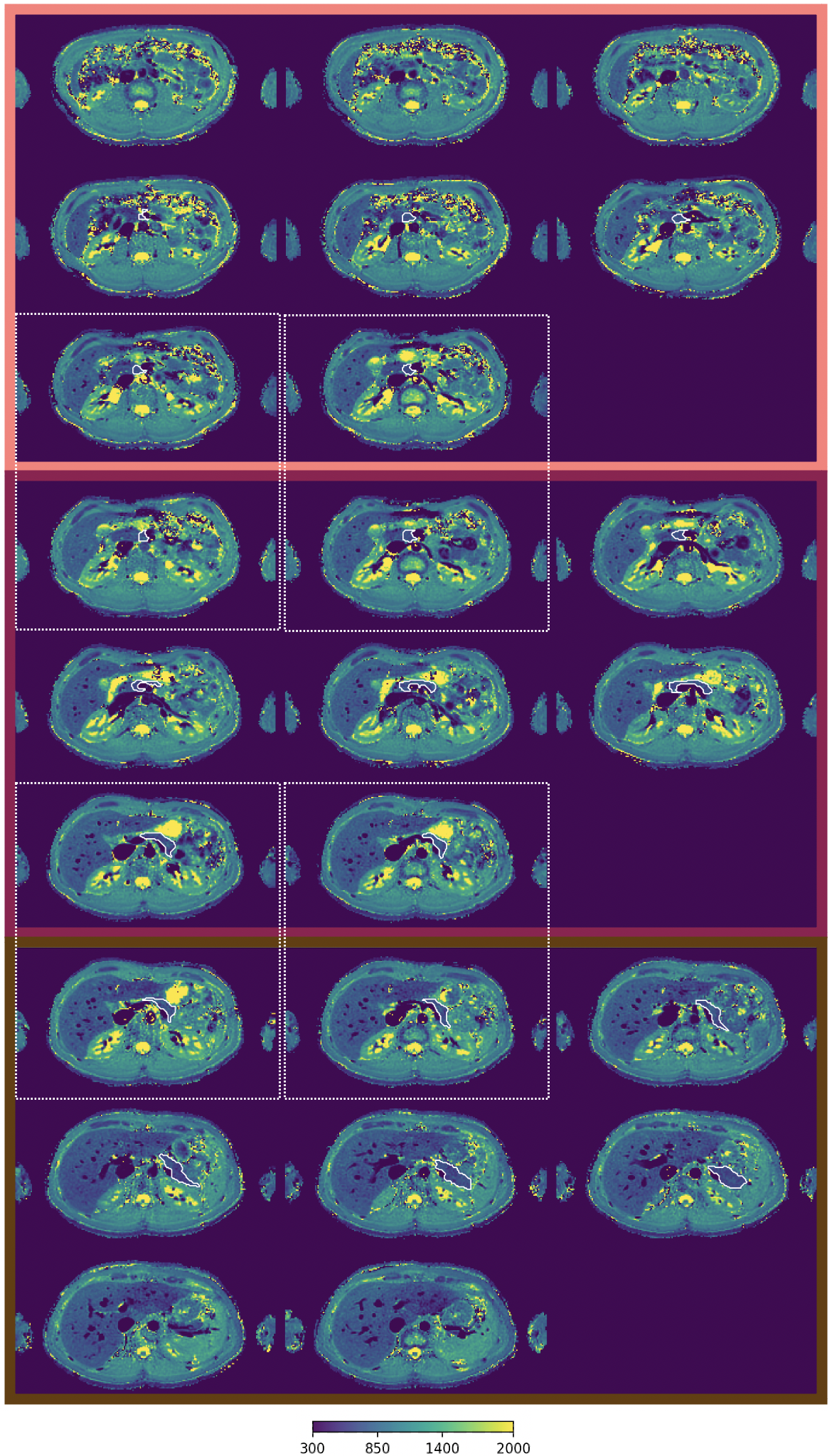
**

**Fig. S2** **Proposed wT_1_ maps in a volunteer.** wT_1_ maps of the whole pancreas. Three stacks of eight slices were acquire for whole pancreas coverage. Each stack, acquired in a breath-hold, is presented in a color box. The pancreas is surrounded by a contour in white on each slice. Dotted white boxes group vertically the corresponding slices in an overlap which show similar anatomical structures.


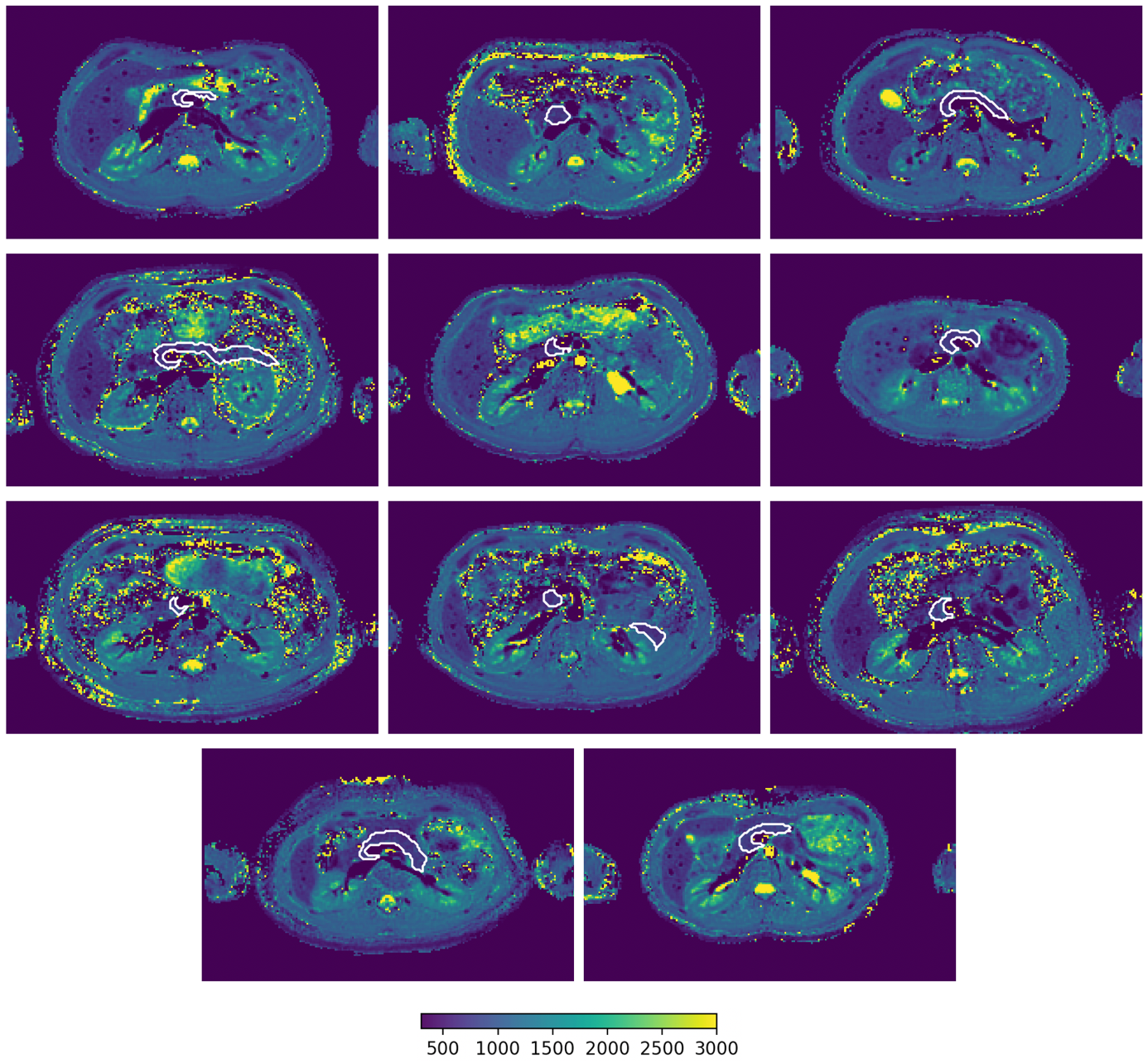


**Fig. S3 Proposed wT**_1_ **map of the middle slice for all volunteers.** The slice four of the second stack is displayed for the 11 volunteers. The corresponding manual segmentation of the pancreas is shown in white. The pancreas parenchyma in the slice presents different shapes among the volunteers.


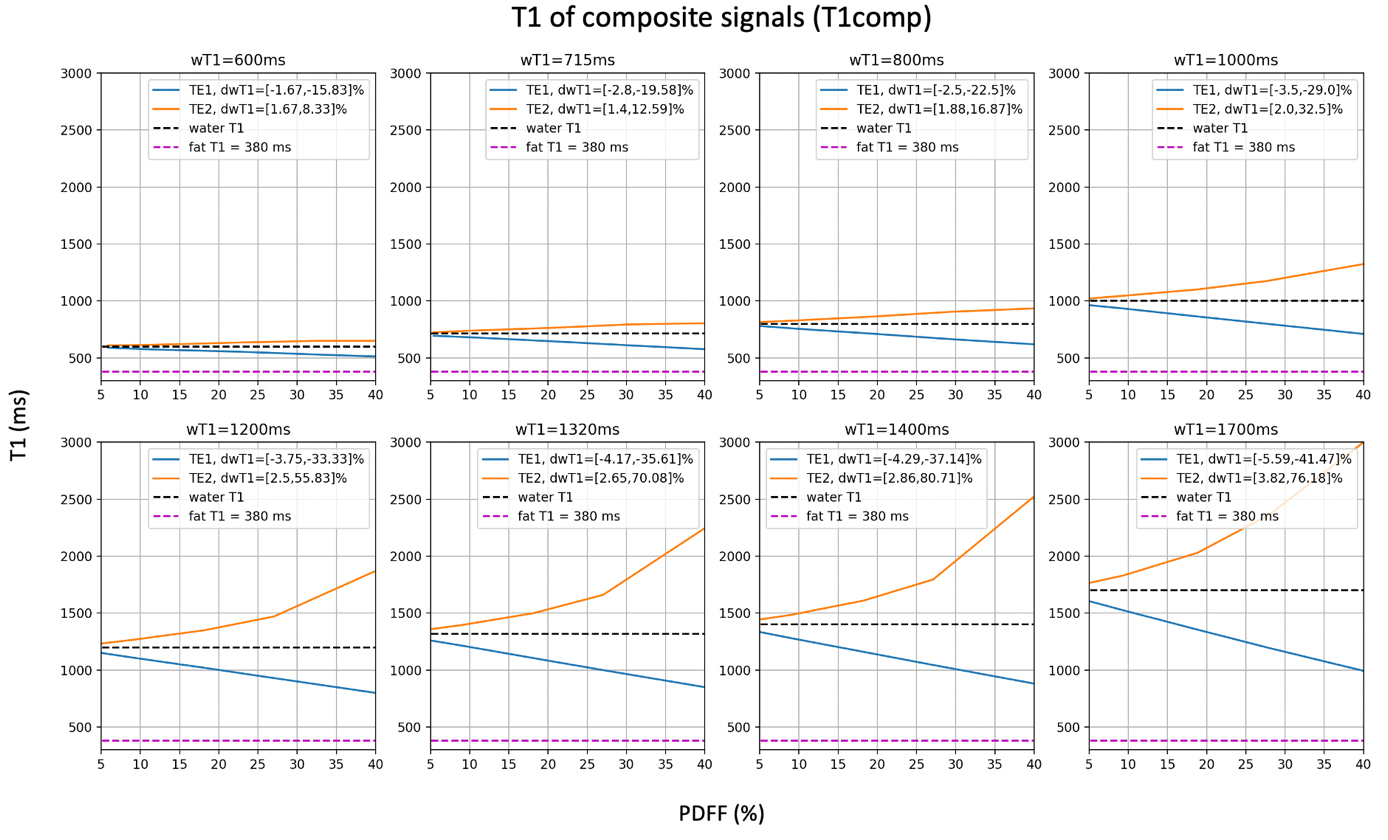


**Fig. S4 Composite signal simulations.** Composite signal simulations for wT1=[600, 715, 800, 1000, 1200, 1320, 1400, 1700] ms, PDFF = [5, 10, 20, 30, 40]%, $B_{1, fraction}^{+}$= [1.0] and TE1/TE2 = 2.3/3.3 ms. For every wT1, a water signal was defined as the dictionary entry with wT1 (dotted black) and T2 = 50ms while the fat signal, as the entry with T1 = 380ms (dotted magenta) and T2 = 50ms. Water and fat signals were mixed (composite signal) according to the PDFF values for TE1 and TE2. Then, the composite signal of each TE was matched to the pre-defined dictionary. The estimated T1comp of the simulated signals for TE1 (blue) and TE2 (orange) are plotted per wT1 against the PDFF used for the simulation.


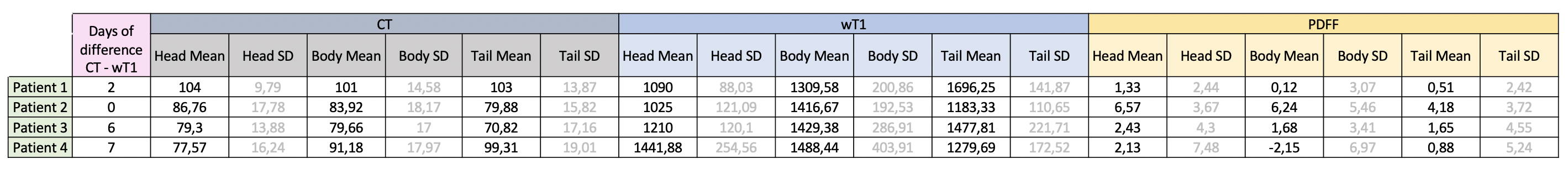

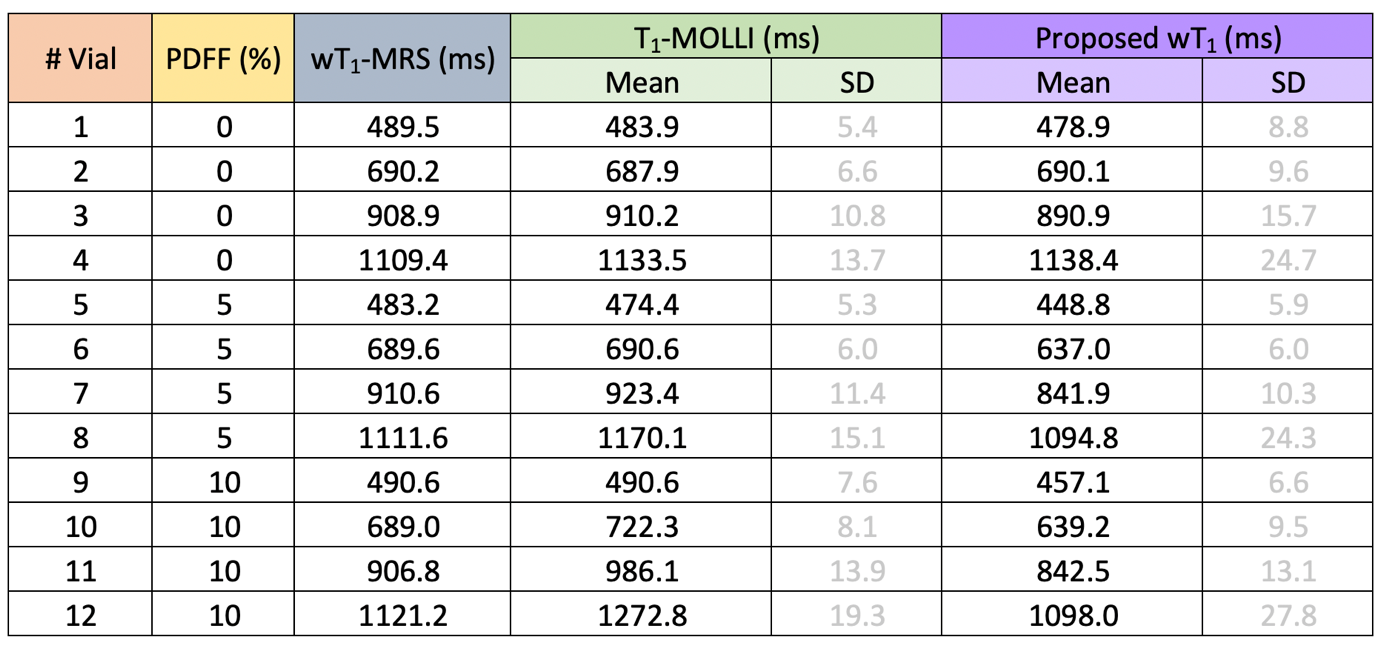


**Table S1 Phantom’s ROI analysis**. Results of the MRS measurements and ROI analysis in the T_1_-MOLLI and the proposed wT_1_ maps for all the vials.

|  |  | Proposed wT_1_ (ms) | | | |
| --- | --- | --- | --- | --- | --- |
|  |  | Measurement 1 | | Measurement 2 | |
|  |  | Mean | SD | Mean | SD |
| Volunteer 1 | Stack 1 | 700.8 | 120.1 | 723.7 | 115.6 |
|  | Stack 2 | 734.5 | 167.4 | 732.3 | 148.0 |
|  | Stack 3 | 697.7 | 118.0 | 684.2 | 119.6 |
| Volunteer 2 | Stack 1 | 745.9 | 98.8 | 710.6 | 120.1 |
|  | Stack 2 | 719.0 | 111.2 | 706.3 | 120.3 |
|  | Stack 3 | 713.9 | 93.9 | 718.7 | 99.5 |
| Volunteer 3 | Stack 1 | 621.4 | 112.7 | 628.3 | 123.6 |
|  | Stack 2 | 604.7 | 125.3 | 618.4 | 164.9 |
|  | Stack 3 | 676.3 | 203.2 | 580.7 | 158.4 |
| Volunteer 4 | Stack 1 | 728.5 | 210.2 | 736.0 | 219.0 |
|  | Stack 2 | 766.7 | 248.3 | 769.2 | 229.1 |
|  | Stack 3 | 770.4 | 188.1 | 759.5 | 228.7 |
| Volunteer 5 | Stack 1 | 654.5 | 147.7 | 633.3 | 114.1 |
|  | Stack 2 | 723.2 | 214.2 | 692.8 | 232.0 |
|  | Stack 3 | 678.8 | 158.0 | 663.6 | 190.2 |
| Volunteer 6 | Stack 1 | 740.3 | 191.0 | 680.2 | 209.9 |
|  | Stack 2 | 693.3 | 176.8 | 699.6 | 220.0 |
|  | Stack 3 | 716.9 | 139.3 | 707.6 | 168.2 |
| Volunteer 7 | Stack 1 | 633.6 | 158.2 | 696.3 | 146.0 |
|  | Stack 2 | 656.1 | 130.3 | 678.8 | 128.7 |
|  | Stack 3 | 658.2 | 132.8 | 641.0 | 148.3 |
| Volunteer 8 | Stack 1 | 732.8 | 110.8 | 768.1 | 124.8 |
|  | Stack 2 | 772.1 | 125.1 | 784.3 | 130.8 |
|  | Stack 3 | 798.8 | 121.6 | 823.0 | 139.7 |
| Volunteer 9 | Stack 1 | 748.9 | 250.7 | 809.4 | 206.0 |
|  | Stack 2 | 718.1 | 232.0 | 739.0 | 245.4 |
|  | Stack 3 | 714.8 | 222.2 | 715.1 | 228.2 |
| Volunteer 10 | Stack 1 | 757.2 | 180.7 | 769.0 | 176.5 |
|  | Stack 2 | 666.5 | 162.5 | 690.4 | 156.1 |
|  | Stack 3 | 755.7 | 146.7 | 748.7 | 157.6 |
| Volunteer 11 | Stack 1 | 766.9 | 150.6 | 739.4 | 173.0 |
|  | Stack 2 | 731.4 | 161.0 | 762.1 | 146.6 |
|  | Stack 3 | 758.1 | 156.8 | 765.7 | 144.4 |

**Table S2 ROI values of two measurements of the proposed wT_1_ maps in volunteers.** The mean and SD of the two consecutive measurements are listed for every stack and volunteer.

|  | T_1_-MOLLI (ms) | | Proposed wT_1_ (ms) | |
| --- | --- | --- | --- | --- |
|  | Mean | SD | Mean | SD |
| Volunteer 1 | 900.1 | 192.8 | 769.2 | 184.4 |
| Volunteer 2 | 856.1 | 111.3 | 720.9 | 147.4 |
|  | 820.1 | 147.6 | 675.7 | 175.7 |
| Volunteer 3 | 839.9 | 231.1 | 549.8 | 113.7 |
|  | 848.0 | 144.5 | 668.3 | 115.5 |
| Volunteer 4 | 1143.7 | 592.7 | 830.1 | 252.8 |
| Volunteer 5 | 897.5 | 111.7 | 623.3 | 142.8 |
|  | 980.9 | 264.4 | 704.5 | 119.1 |
| Volunteer 6 | 1088.4 | 671.0 | 662.4 | 169.9 |
|  | 1095.2 | 201.9 | 692.8 | 186.0 |
|  | 1148.4 | 210.8 | 727.7 | 194.2 |
| Volunteer 7 | 936.7 | 232.7 | 640.2 | 104.7 |
|  | 899.6 | 205.7 | 679.2 | 166.7 |
| Volunteer 8 | 987.6 | 140.8 | 799.5 | 100.7 |
|  | 924.5 | 123.7 | 789.5 | 133.4 |
| Volunteer 9 | 1129.0 | 492.7 | 749.5 | 222.8 |
|  | 1115.0 | 247.9 | 819.3 | 225.6 |
| Volunteer 10 | 1016.6 | 191.5 | 700.6 | 127.6 |
|  | 1021.6 | 223.1 | 741.9 | 147.3 |
| Volunteer 11 | 1009.8 | 132.7 | 767.6 | 174.2 |
|  | 1024.1 | 134.0 | 796.8 | 115.9 |

**Table S3 T_1_-MOLLI vs. the proposed wT_1_ in volunteers.** The mean and SD of the ROIs defined in the T_1_-MOLLI maps and in their corresponding wT_1_ maps are displayed. Up to three T_1_-MOLLI maps were acquired per volunteer indistinctly.

|  | CT (HU) | | | | | |
| --- | --- | --- | --- | --- | --- | --- |
|  | Head Mean | Head SD | Body Mean | Body SD | Tail Mean | Tail SD |
| Patient 1 | 104.0 | 9.8 | 101.0 | 14.6 | 103.0 | 13.9 |
| Patient 2 | 86.8 | 17.8 | 83.9 | 18.2 | 79.9 | 15.8 |
| Patient 3 | 79.3 | 13.9 | 79.7 | 17.0 | 70.8 | 17.2 |
| Patient 4 | 77.6 | 16.2 | 91.2 | 18.0 | 99.3 | 19.0 |
|  |  |  |  |  |  |  |
|  | Proposed wT_1_ (ms) | | | | | |
|  | Head Mean | Head SD | Body Mean | Body SD | Tail Mean | Tail SD |
| Patient 1 | 1090.0 | 88.0 | 1309.6 | 200.9 | 1696.3 | 141.9 |
| Patient 2 | 830.56 | 121.1 | 1416.7 | 192.5 | 1183.3 | 110.7 |
| Patient 3 | 1210.0 | 120.1 | 1429.4 | 286.9 | 1477.8 | 221.7 |
| Patient 4 | 1441.9 | 254.6 | 1488.4 | 403.9 | 1279.7 | 172.5 |
|  |  |  |  |  |  |  |
|  | PDFF (%) | | | | | |
|  | Head Mean | Head SD | Body Mean | Body SD | Tail Mean | Tail SD |
| Patient 1 | 1.3 | 2.4 | 0.1 | 3.1 | 0.5 | 2.4 |
| Patient 2 | 6.6 | 3.7 | 6.2 | 5.5 | 4.2 | 3.7 |
| Patient 3 | 2.4 | 4.3 | 1.7 | 3.4 | 1.7 | 4.6 |
| Patient 4 | 2.1 | 7.5 | -2.2 | 7.0 | 0.9 | 5.2 |

|  | Time difference in the acquisition of CT and wT_1_ (Days) |
| --- | --- |
|  |  |
| Patient 1 | 2 |
| Patient 2 | 0 |
| Patient 3 | 6 |
| Patient 4 | 7 |

**Table S4 ROI analysis in patients.** The mean and SD of the ROIs defined in the head, body and tail of the pancreas in the CT images, proposed wT_1_ and PDFF maps are shown in the first three tables. The CT images were acquired days before the proposed wT_1_ in some cases. The last table shows the time difference in the acquisition of these images.
